# Trends in types of protein in US adolescents and children: Results from the National Health and Nutrition Examination Survey 1999-2010

**DOI:** 10.1371/journal.pone.0230686

**Published:** 2020-03-26

**Authors:** Hyunju Kim, Laura E. Caulfield, Casey M. Rebholz, Rebecca Ramsing, Keeve E. Nachman

**Affiliations:** 1 Center for Human Nutrition, Johns Hopkins Bloomberg School of Public Health, Baltimore, Maryland, United States of America; 2 Welch Center for Prevention, Epidemiology and Clinical Research, Johns Hopkins Bloomberg School of Public Health, Baltimore, Maryland, United States of America; 3 Center for a Livable Future, Johns Hopkins Bloomberg School of Public Health, Baltimore, Maryland, United States of America; 4 Department of Epidemiology, Johns Hopkins Bloomberg School of Public Health, Baltimore, Maryland, United States of America; 5 Department of Environmental Health and Engineering, Johns Hopkins Bloomberg School of Public Health, Baltimore, Maryland, United States of America; 6 Department of Health Policy and Management, Johns Hopkins Bloomberg School of Public Health, Baltimore, Maryland, United States of America; Yeungnam University, REPUBLIC OF KOREA

## Abstract

**Background:**

It is unclear if the intakes of different types of protein have changed over time.

**Objective:**

We delineated trends in types of protein (beef, pork, lamb or goat, chicken, turkey, fish, dairy, eggs, legumes, and nuts and seeds) in US children (2-<12 years) and adolescents (12–19 years) from 1999 to 2010.

**Methods:**

We used 6 repeated cross-sectional surveys (National Health and Nutrition Examination Survey 1999–2010, n≥1,665 for children; n≥1,156 for adolescents) to test for linear trends in the intake of types of protein (grams per kilogram of body weight) among children and adolescents, and according to sociodemographic groups and participation in food assistance programs.

**Results:**

Among children, pork intake (0.76 to 0.51 g/kg) decreased, but chicken (0.98 to 1.28 g/kg), all poultry (1.18 to 1.55 g/kg), egg (0.63 to 0.69 g/kg), and legume (0.35 to 0.54 g/kg) intake increased (all *P*<0.05). Among adolescents, beef intake decreased (0.92 to 0.67 g/kg) whereas chicken (0.59 to 0.74 g/kg) and all poultry (0.72 to 0.86 g/kg) intake increased from 1999 to 2010 (all *P*<0.01). Participants of the Women, Infants, and Children (WIC) increased the intake of chicken and dairy (all *P*<0.05) over time whereas no significant trend was observed for income-eligible non-participants. Fish intake did not change in any age group, and recommended types of protein (poultry, fish, nuts and seeds) declined among children of lower socioeconomic status.

**Conclusions:**

Intake of recommended types of protein increased among children, adolescents and WIC participants. However, subgroup analyses suggest socioeconomic disparities.

## Introduction

Research has suggested that eating habits developed in childhood track into adulthood, and these dietary behaviors may influence the risk of chronic diseases [1–4]. Therefore, there has been an increasing recognition that it is important to establish healthy eating habits earlier in life for future optimal health.

Of several dietary components, protein intake is important for the period of development and growth and health status in later life [5]. It has been reported that most children and adolescents in the US consume more protein than needed to meet requirements. However, a recent study found that adolescent girls fall short of meeting the recommended amount of protein, with almost 25% reporting protein intakes below the recommended dietary allowances [6]. Considering that lean meats, fish, and plant proteins are encouraged as dietary sources of protein for children and adolescents [7], and the wide range of sources of protein (beef, pork, chicken, legumes etc) available, data on intakes of protein foods would be helpful in identifying the types of protein that can be promoted among those with inadequate intake. Currently, there are no published reports of intakes of different types of protein for children and adolescents.

Further, no studies have examined whether intakes of types of protein have changed over time. Since 2000, food and nutrition policies have evolved to foster healthy eating habits in children and adolescents, which could have affected dietary intakes, specifically protein food intakes. For example, the Dietary Guidelines, which provide recommendations for individuals ≥2 y, have emphasized the intake of seafood and plant proteins over time [7–10]. Changes in the Special Supplemental Nutrition Program for Women, Infant, and Children (WIC) have occurred as well, with the revision of its food packages to include lower fat dairy instead of full-fat dairy to align with the Dietary Guidelines [8,11]. The Child Nutrition Act, reauthorized in 2004, has continued to provide nutritious meals for free or at a reduced cost for low-income children [12]. In addition, the number of participants in Supplemental Nutrition Assistance Program (SNAP), which enables low-income individuals to purchase diverse protein foods, has more than doubled from 1999 to 2012 [13], suggesting that there may be changes in the intake of meats, poultry, fish, and dairy products among low-income families over time.

There have been earlier studies of dietary intakes of US children and adolescents over time, but they examined composite measures such as diet quality or focused only on total protein intake [6,13]. We aimed to address gaps in the literature by providing more comprehensive estimates of change in intake of protein over time among children and adolescents by focusing on different types of protein (beef, pork, chicken, turkey, seafood, dairy, eggs as animal sources of protein, and legumes, and nuts and seeds as plant sources of protein). We also investigated whether these trends differ by population subgroup (sex, race/ethnicity, income, number of people in household, and participation in federal food assistance programs).

## Methods

### Study design

We used dietary intake data from children (2-<12 y) and adolescents (12–19 y) in 6 repeated cross-sectional surveys of the National Health and Nutrition Examination Survey (NHANES). NHANES collects data on the health and nutritional status of the US civilian population biennially. Participants attended health examinations at a mobile examination center, where they are asked to report all foods and beverages consumed over the past 24 hours [14]. For children aged <6 y, household members reported the child’s food and beverage intake [15]. For those aged 6-<12 y, children provided their own data with the assistance from a household member [15]. For those aged 12-<20y, adolescents completed the dietary recall on their own [15].

In all cycles, we used participants’ first day of 24 hour dietary recall given that the earlier survey cycles (1999–2000 and 2001–2002) did not collect a second dietary recall, and to maximize the number of survey cycles included in the analyses. The total analytic sample was 22,464 (children: n = 11,251; adolescents: n = 11,213), and the analytic sample for each survey cycle ranged from 1,665 to 2,020 for children and 1,156 to 2,296 for adolescents.

### Types of protein

We used the Food Commodity Intake Databases (FCID) and its recipe databases to disaggregate all food items reported in the 24 hour dietary recalls with respect to type of protein [16]. Classification of commodity items to different types of protein have been reported in our previous paper [17].

We focused on the following types of protein: beef, pork, lamb or goat, chicken, turkey, all poultry, fish and shellfish, milk and milk products (dairy), eggs, legumes, and nuts and seeds. We did not study the trends in sub-classes of dairy (full-fat, low-fat, or non-fat products) or processed meats because FCID did not have these data. To be consistent with how protein is recommended in the Dietary Reference Intakes (DRIs), the data are presented in grams of intake (g) per kilogram (kg) of body weight.[18]

### Population subgroups

We assessed whether trends in types of protein differed by sex (boys, girls), race/ethnicity (non-Hispanic white, non-Hispanic black, Hispanic, or other race), income (a ratio of family income to poverty threshold (PIR): ≤1.30, 1.31-<3.50, ≥3.50), number of individuals in a household (<3, ≥3), and participation in food assistance programs: SNAP (yes/no), WIC (yes/no), and National School Lunch Program and the School Breakfast Program (NSLP/SBP) (yes/no).

To characterize participation in federal food assistance programs, we used the same income and age cut-offs as previous studies [13,19]. SNAP participation status was classified based on self-reports of receiving household SNAP benefits in the past year. Individuals with PIR≤1.30 who did not report receiving SNAP benefits were considered income-eligible non-participants. WIC participation status was classified based on self-reports of the child (1-<5 y) receiving WIC benefits in the past year. Subjects with PIR≤1.85 who did not report receiving WIC benefits were considered income-eligible non-participants. NSLP/SBP participation status was also classified based on self-reports of receiving either free or reduced-priced meals at schools. Those children aged 5–18 y with PIR≤1.85 who did not report receiving free or reduced-priced meals were considered income-eligible non-participants.

### Statistical analyses

We examined trends in protein food intake in the overall study population (2-<12 y, and 12–19 y) and by age (2-<6 y, 6-<12 y, and ≥12 y) in terms of: 1) mean intake (g/kg) in the overall study population in each survey cycle, 2) proportion of children or adolescents consuming more than 0 grams of a protein food on a given day (henceforth referred to as “consumers”), and 3) mean intake of each protein food (g/kg) among consumers only. We used survey-weighted linear regression models to test for a linear trend using survey cycles as an ordinal variable (from 1999 to 2010).

For subgroup analyses, we examined only the mean intake of protein foods. We tested whether the trends in the types of protein differed across subgroups using cross-product terms between survey cycles and categorical variables (sex, race/ethnicity, income, number of people in a household, participation in the federal food assistance programs).

As sensitivity analyses, we 1) repeated our analyses by additionally adjusting for age, 2) adjusting for total energy intake instead of body weight, and 3) restricting the data to NHANES 2003 to 2010 when two days of dietary recalls were available and we used the average intake of the two-day dietary recalls. We considered an alpha level <0.05 to indicate statistically significant trends, and <0.1 to indicate statistically significant interactions. We used 0.1 for *P* interaction given that we may have been underpowered to detect differences in trends in subgroups due to smaller sample sizes. Analyses were conducted in 2018 using Stata version 13.0 (StataCorp, College Station, TX).

## Results

### Mean intake of types of protein in the overall study population

In all age groups, total meat consumption (beef, pork, lamb or goat, chicken, turkey, and fish) did not change from 1999 to 2010 (all *P* trend>0.05), but daily beef or pork intake declined when stratified by age (2-<6 y, beef: 1.66 to 1.33 g/kg; 6-<12 y, pork: 0.76 to 0.51 g/kg; 12-<20 y, beef: 0.92 to 0.67 g/kg; all *P* trend<0.05) (Table 1, S1 Table). Among children, chicken (0.98 to 1.28 g/kg), all poultry (1.18 to 1.55 g/kg), eggs (0.63 to 0.69 g/kg), and legume intake (0.35 to 0.54 g/kg, *P* = 0.01) increased significantly. These trends (specifically chicken and all poultry) were also observed among adolescents (all *P*<0.05).

**Table 1:** Mean intake of types of protein in US adolescents and children.

	1999–2000	2001–2002	2003–2004	2005–2006	2007–2008	2009–2010	Percent change^2^	
	Intake in grams per kg of body weight (g/kg) ± SE for the overall study population^1^							*P* trend
	2-<12 years of age							
Sample size	1,701	1,995	1,665	1,917	1,953	2,020		
Beef	1.38 ± 0.12	1.28 ± 0.07	1.35 ± 0.07	1.08 ± 0.06	1.34 ± 0.09	1.13 ± 0.06	-18.1	0.08
Pork	0.75 ± 0.07	0.57 ± 0.03	0.59 ± 0.03	0.68 ± 0.06	0.61 ± 0.02	0.57 ± 0.03	-24.0	0.15
Lamb or goat	0.009 ± 0.002	0.01 ± 0.007	0.02 ± 0.007	0.02 ± 0.006	0.01 ± 0.006	0.01 ± 0.01	11.1	0.946
Chicken	0.98 ± 0.06	0.89 ± 0.09	1.17 ± 0.04	1.11 ± 0.05	1.2 ± 0.06	1.28 ± 0.06	30.6	<0.001
Turkey	0.20 ± 0.03	0.22 ± 0.02	0.26 ± 0.03	0.21 ± 0.009	0.27 ± 0.03	0.25 ± 0.01	25.0	0.09
All poultry	1.18 ± 0.07	1.10 ± 0.1	1.43 ± 0.03	1.32 ± 0.06	1.47 ± 0.07	1.55 ± 0.06	31.4	<0.001
Fish and shellfish	0.18 ± 0.03	0.27 ± 0.06	0.19 ± 0.03	0.28 ± 0.07	0.19 ± 0.01	0.21 ± 0.05	16.7	0.89
Milk and Milk products	17.23 ± 0.86	17.82 ± 0.71	18.96 ± 0.78	17.84 ± 0.51	17.33 ± 0.53	18.68 ± 0.38	8.4	0.43
Eggs	0.63 ± 0.04	0.59 ± 0.05	0.68 ± 0.06	0.72 ± 0.05	0.72 ± 0.03	0.69 ± 0.04	9.5	0.04
Legumes	0.35 ± 0.04	0.35 ± 0.06	0.59 ± 0.13	0.60 ± 0.11	0.65 ± 0.08	0.54 ± 0.08	54.3	0.03
Nuts and Seeds	0.45 ± 0.05	0.35 ± 0.03	0.41 ± 0.06	0.35 ± 0.03	0.31 ± 0.03	0.41 ± 0.03	-8.9	0.27
	12–19 years of age							
Sample Size	2,219	2,296	2,162	2,115	1,156	1,265		
Beef	0.92 ± 0.09	0.79 ± 0.05	0.89 ± 0.04	0.86 ± 0.04	0.74 ± 0.04	0.67 ± 0.06	-27.2	0.01
Pork	0.34 ± 0.03	0.42 ± 0.05	0.37 ± 0.04	0.37 ± 0.02	0.34 ± 0.03	0.41 ± 0.03	20.6	0.93
Lamb or goat	0.01 ± 0.01	0.01 ± 0.01	0.02 ± 0.01	0.005 ± 0.001	0.006 ± 0.005	0.02 ± 0.005	100.0	0.84
Chicken	0.59 ± 0.05	0.56 ± 0.03	0.60 ± 0.03	0.68 ± 0.04	0.71 ± 0.04	0.74 ± 0.05	25.4	0.002
Turkey	0.13 ± 0.02	0.12 ± 0.01	0.15 ± 0.01	0.15 ± 0.01	0.17 ± 0.03	0.12 ± 0.01	-7.7	0.34
All poultry	0.72 ± 0.05	0.68 ± 0.03	0.75 ± 0.03	0.83 ± 0.03	0.88 ± 0.05	0.86 ± 0.05	19.4	0.001
Fish and shellfish	0.11 ± 0.01	0.09 ± 0.01	0.10 ± 0.02	0.10 ± 0.02	0.11 ± 0.02	0.12 ± 0.01	9.1	0.72
Milk and Milk products	5.64 ± 0.34	6.13 ± 0.36	5.97 ± 0.38	5.2 ± 0.25	5.13 ± 0.32	5.31 ± 0.32	-5.9	0.09
Eggs	0.28 ± 0.01	0.35 ± 0.02	0.26 ± 0.01	0.29 ± 0.01	0.34 ± 0.02	0.32 ± 0.02	14.3	0.38
Legumes	0.13 ± 0.03	0.14 ± 0.02	0.13 ± 0.01	0.13 ± 0.02	0.14 ± 0.02	0.16 ± 0.03	23.1	0.56
Nuts and Seeds	0.17 ± 0.02	0.18 ± 0.02	0.23 ± 0.03	0.20 ± 0.02	0.21 ± 0.02	0.21 ± 0.02	23.5	0.16

^1^ g/kg indicates grams of protein food intake per kilogram of body weight, and SE indicates standard errors.

^2^ Percent change from 1999–2000 to 2009–2010

### Percent of consumers and mean intake of types of protein among consumers

The trends in consumers were similar to the trends in the overall study population. For instance, the proportion of children consuming chicken (46 to 54%) and all poultry (52 to 58%) increased from 1999 to 2010 (all *P*<0.001, Table 2). The proportion of adolescents consuming beef on a given day decreased significantly (74 to 67%, *P* = 0.02), whereas chicken, turkey, all poultry, and dairy consumers increased significantly (all *P*<0.05) (Table 2).

**Table 2:** Proportion of adolescents and children consuming different types of protein and mean intake among consumers.

	1999–2000	2001–2002	2003–2004	2005–2006	2007–2008	2009–2010	Percent change^2^	
	2-<12 years of age: Percent of Consumers, %^1^							*P*-trend
Beef	73	74	78	70	76	72	-1.4	0.51
Pork	63	62	64	61	65	59	-6.3	0.53
Lamb or goat	5	7	7	5	6	5	0.0	0.704
Chicken	46	43	51	53	53	54	17.4	<0.001
Turkey	23	22	23	22	27	26	13.0	0.052
All poultry	52	47	55	56	57	58	11.5	<0.001
Fish and shellfish	9	10	10	13	9	10	11.1	0.64
Milk and Milk products	98	94	99	99	99	99	1.0	<0.001
Eggs	82	79	86	85	86	82	0.0	0.19
Legumes	70	66	73	69	69	67	-4.3	0.85
Nuts and Seeds	79	73	79	79	78	78	-1.3	0.16
	2-<12 years of age: Intake in grams of per kg of body weight (g/kg) ± SE among consumers only^2^							
Beef	1.89 ± 0.15	1.73 ± 0.07	1.74 ± 0.11	1.54 ± 0.07	1.75 ± 0.1	1.57 ± 0.06	-16.9	0.09
Pork	1.20 ± 0.10	0.92 ± 0.04	0.93 ± 0.07	1.11 ± 0.07	0.95 ± 0.03	0.96 ± 0.06	-20.0	0.19
Lamb or goat	0.21 ± 0.04	0.17 ± 0.11	0.30 ± 0.18	0.29 ± 0.14	0.19 ± 0.09	0.25 ± 0.12	19.0	0.83
Chicken	2.11 ± 0.06	2.05 ± 0.14	2.3 ± 0.13	2.11 ± 0.10	2.25 ± 0.09	2.38 ± 0.12	12.8	0.04
Turkey	0.89 ± 0.07	0.98 ± 0.10	1.12 ± 0.15	0.95 ± 0.06	1.00 ± 0.08	0.97 ± 0.08	9.0	0.70
All poultry	2.27 ± 0.06	2.32 ± 0.15	2.61 ± 0.10	2.36 ± 0.10	2.58 ± 0.12	2.65 ± 0.10	16.7	0.005
Fish and shellfish	2.05 ± 0.18	2.65 ± 0.33	1.97 ± 0.31	2.19 ± 0.33	2.15 ± 0.22	2.03 ± 0.26	-1.0	0.45
Milk and Milk products	17.37 ± 0.87	18.9 ± 0.71	19.02 ± 0.81	17.87 ± 0.41	17.36 ± 0.62	18.72 ± 0.55	7.8	0.91
Eggs	0.77 ± 0.05	0.74 ± 0.05	0.80 ± 0.06	0.85 ± 0.05	0.84 ± 0.04	0.84 ± 0.04	9.1	0.11
Legumes	0.50 ± 0.06	0.53 ± 0.09	0.81 ± 0.22	0.87 ± 0.16	0.94 ± 0.17	0.80 ± 0.11	60.0	<0.001
Nuts and Seeds	0.58 ± 0.06	0.48 ± 0.04	0.51 ± 0.05	0.45 ± 0.03	0.39 ± 0.03	0.52 ± 0.04	-10.3	0.16
	12–19 years of age: Percent of Consumers, %							
Beef	74	72	76	76	68	67	-9.5	0.02
Pork	59	63	60	63	56	58	-1.7	0.16
Lamb or goat	7	8	6	5	5	7	0	0.151
Chicken	47	46	48	49	54	52	10.6	0.02
Turkey	22	22	22	22	28	25	13.6	0.048
All poultry	50	50	52	52	57	56	12.0	0.02
Fish and shellfish	10	9	10	9	11	12	20.0	0.13
Milk and Milk products	98	97	99	99	99	99	1.0	<0.001
Eggs	82	80	82	82	80	81	-1.2	0.86
Legumes	57	60	62	61	62	60	5.3	0.29
Nuts and Seeds	76	77	76	77	72	76	0	0.34
	12–19 years of age: Intake in grams of per kg of body weight (g/kg) ± SE among consumers only^3^							
Beef	1.24 ± 0.09	1.10 ± 0.05	1.17 ± 0.06	1.14 ± 0.03	1.09 ± 0.06	1.00 ± 0.07	-19.4	0.05
Pork	0.58 ± 0.03	0.67 ± 0.07	0.61 ± 0.04	0.59 ± 0.04	0.61 ± 0.05	0.71 ± 0.09	22.4	0.53
Lamb or goat	0.16 ± 0.09	0.19 ± 0.07	0.26 ± 0.12	0.10 ± 0.02	0.11 ± 0.09	0.26 ± 0.16	62.5	0.92
Chicken	1.26 ± 0.05	1.21 ± 0.04	1.25 ± 0.08	1.37 ± 0.08	1.32 ± 0.05	1.42 ± 0.07	12.7	0.01
Turkey	0.57 ± 0.06	0.55 ± 0.03	0.66 ± 0.06	0.71 ± 0.05	0.63 ± 0.14	0.50 ± 0.04	-12.3	0.89
All poultry	1.43 ± 0.05	1.36 ± 0.04	1.44 ± 0.08	1.59 ± 0.08	1.55 ± 0.09	1.55 ± 0.07	8.4	0.02
Fish and shellfish	1.10 ± 0.10	1.15 ± 0.09	1.08 ± 0.10	1.15 ± 0.07	0.97 ± 0.15	1.02 ± 0.14	-7.3	0.32
Milk and Milk products	5.75 ± 0.34	6.33 ± 0.39	6.04 ± 0.39	5.26 ± 0.31	5.19 ± 0.30	5.35 ± 0.40	-7.0	0.05
Eggs	0.35 ± 0.02	0.44 ± 0.02	0.32 ± 0.02	0.35 ± 0.02	0.42 ± 0.04	0.40 ± 0.04	14.3	0.39
Legumes	0.23 ± 0.05	0.23 ± 0.03	0.21 ± 0.03	0.22 ± 0.02	0.22 ± 0.03	0.27 ± 0.04	17.4	0.68
Nuts and Seeds	0.22 ± 0.02	0.23 ± 0.02	0.31 ± 0.03	0.26 ± 0.04	0.29 ± 0.04	0.28 ± 0.04	27.3	0.11

^1^ Consumers are defined as those who consumed a specific type of protein more than 0 grams a day.

^2^ Percent change from 1999–2000 to 2009–2010

^3^ g/kg indicates grams of protein food intake per kilogram of body weight, and SE indicates standard errors.

### Population subgroups

We observed differences in trends in protein food intake by sex, race/ethnicity, and socioeconomic status. Fish and egg intake increased for girls, but not boys (all *P* interaction<0.1, S3 Table). Among white children, nuts and seeds showed a decreasing trend (S4 Table). Children from a lower-income household or household size of <3 people decreased the intake of fish, nuts and seeds, or poultry (all *P* interaction<0.1, S5 and S6 Tables).

Interestingly, adolescents showed different patterns from children. Fish and nuts and seeds intake increased more for those who are white compared to others (all *P* interaction<0.05). However, dairy and egg intake declined among adolescents from a higher-income household, and no significant difference was observed by household size.

We found the most consistent difference in trends by WIC participation status (1- <5 y). Among WIC participants, intakes of several recommended types of protein such as chicken (participants: 1.24 to 1.87 g/kg; non-participants: 1.64 to 1.30 g/kg) and dairy (participants: 24.6 to 30.2 g/kg; non-participants: 29.5 to 26.9 g/kg) increased, but these trends were not observed among income-eligible non-participants (Table 3). Furthermore, intake of fish and eggs declined less among WIC participants compared to their income-eligible counterparts (all *P* interaction<0.1).

**Table 3:** Mean intake of types of protein stratified by WIC participation (1-<5 years).

	WIC participants			Income-eligible non-participants			
	Intake in grams of protein foods (g) per kg of body weight ± SE^1^						
	1999–2000	2009–2010	Percent change^2^	1999–2000	2009–2010	Percent change^2^	
	(n = 533)	(n = 662)		(n = 183)	(n = 167)		
	Children (1-<5 years of age)						*P* interaction
Beef	1.58 ± 0.19	1.35±0.11	-14.6	1.53 ± 0.28	1.27±0.17	-17.0	0.25
Pork	0.74 ± 0.12	0.63±0.07	-14.9	0.67 ± 0.12	0.50±0.08	-25.4	0.37
Lamb or goat	0.04 ± 0.02	0.01±0.01	-75.0	0.01 ± 0.01	0.01±0.003	0	0.50
Chicken	1.24 ± 0.18	1.87±0.06***	50.8	1.64 ± 0.29	1.30±0.17	-20.7	0.02
Turkey	0.27 ± 0.03	0.29±0.04*	7.4	0.32 ± 0.11	0.45±0.13	40.6	0.16
All Poultry	1.51 ± 0.19	2.18±0.08***	44.4	1.95 ± 0.32	1.76±0.21	-9.7	0.15
Fish and shellfish	0.28 ± 0.14	0.19±0.04	-32.1	0.17 ± 0.05	0.06±0.04	-64.7	0.06
Milk and Milk products	24.6 ± 1.27	30.2±1.29***	22.7	29.5 ± 3.03	26.9±3.29	-8.6	<0.001
Eggs	1.32 ± 0.11	1.11±0.12	-15.9	1.08 ± 0.17	0.59±0.09	-45.4	0.02
Legumes	1.19 ± 0.14	1.26±0.33	5.9	0.67 ± 0.11	0.91±0.17	35.8	0.69
Nuts and Seeds	0.40 ± 0.09	0.44±0.09	10.0	0.42 ± 0.13	0.65±0.13	54.8	0.65

^1^ Linearized standard error

^2^ Percent change from 1999–2000 to 2009–2010

WIC, Women, Infants, and Children.

Asterisks indicate a statistical significance in trends in food sources of protein within a subgroup (* *P* <0.05,** *P*<0.01, *** *P*<0.001)

Turkey intake showed a decreasing trend among SNAP and NSLP/SBP participants, but an increasing trend among income-eligible non-participants (all *P* interaction<0.1) (S7 and S8 Tables). Intake of chicken and fish increased more or declined less for NSLP/SBP participants than income-eligible non-participants.

### Sensitivity analyses

When we adjusted for total energy intake instead of body weight, the decline in adolescents’ beef intake was attenuated (56.9 to 43.5 g, *P*-trend = 0.08, S9 Table). When we restricted the data to 2003 to 2010, changes in chicken, all poultry, eggs, and legume intake in children and beef intake in adolescents were attenuated (*P*-trend>0.05, S10 Table). The results did not change for all other protein foods, or when we additionally adjusted for age.

## Discussion

In this nationally representative sample of children and adolescents, beef or pork intake decreased, whereas chicken intake increased significantly across all age groups from 1999 to 2010. WIC participants showed favorable trends in the intakes of several recommended types of protein, while intakes did not change among income-eligible non-participants. We found no change in fish consumption in the overall study population. In children of lower socioeconomic status, fish and nuts and seeds declined over time. Our data extends results from a recent analysis of protein intake by identifying the specific types of protein that may warrant being promoted, and population subgroups who may be warrant being targeted through programs [6].

Our results are largely consistent with a previous study which examined changes in diet quality of children and adolescents over time. This prior study reported that seafood and plant proteins increased from 1999 to 2012 among children and adolescents. Similar to this previous study, we found an increase in the intake of plant proteins (legumes), but only among children [13]. Slight differences in the results may be due to differences in the time period studied, because our study used data from 1999 to 2010. FCID, which was used to disaggregate foods into different types of protein in our study, was available only until 2010, thus, we could not examine trends in the most recent NHANES cycles [16]. Further, the previous study did not report plant protein intake separately, but rather combined plant proteins with seafood and plant protein; and incorporated plant protein into the total protein intake category, whereas our study calculated changes in the intake of fish, legumes, and nuts separately, providing more detailed data.

Since 2000, US Department of Agriculture (USDA) reported that availability of beef, pork, poultry, and nuts has been steadily increasing, whereas availability of eggs, whole milk, and legumes has been decreasing [20]. These supply side of data have some overlaps with our results on trends in consumption. Specifically, these data were in line with an increase in chicken and poultry intake in NHANES 2003–2004 relative to 1999–2002 in that per capita poultry availability was the highest in 2004, and the availability of broiler (i.e. chicken that is bred for meat production) was higher in 2004 than the early 2000s [21]. Despite the increases in supply of various protein foods, diet quality of US food supply was reported to be low (below 60 out of 100) [22]. In particular, milk and legumes were not supplied enough to meet the dietary recommendation [22], which suggests that that it remains important to improve the quality of protein foods available in the US food supply.

A reduction in beef or pork intake and an increase in recommended types of protein is encouraging in that they align with the Dietary Guidelines to increase the intake of lean meats and plant protein [8–10]. However, we found no change in fish intake and fish consumption did decrease among several groups (boys, blacks, Hispanics, and children from lower-income households). The Dietary Guidelines and American Academy of Pediatrics emphasized that fish provides health benefits for the general population, and for children and adolescents [7,23]. Despite the health benefits and recommendations, concerns about mercury contamination from seafood remain [23]. Thus, fish that are low in mercury such as salmon and sardines were specifically suggested in the most recent Dietary Guidelines [7]. Given these changes, follow-up studies with more recent data on NHANES could inform whether fish intakes are changing.

We observed an increase or less substantial decline in chicken, dairy, fish, and egg intake for WIC participants compared to income-eligible non-participants. Our findings build upon previous studies which have documented positive dietary changes among WIC participants including a higher diet quality and low-fat dairy consumption compared to income-eligible non-participants [11,24,25]. We found a higher poultry intake among WIC participants over time interesting, because WIC packages do not include chicken or turkey. Higher consumption of poultry in this group may be due to the program’s nutrition education component which provides guidance on healthy eating or increasing preference for poultry as a dietary source of protein in general [17].

With regard to participation in other federal food assistance programs, our findings on SNAP concur with a systematic review which showed that protein food intake (meat, beans, milk) did not differ between children who are receiving SNAP benefits and income-eligible non-participants [26]. Studies comparing dietary intakes of NSLP/SBP participants and non-participants are limited, but some reports indicate that participants were more likely to have better nutritional intakes, and have higher intakes of protein as a percentage of energy at lunches than their matched non-participants who were similar with respect to several sociodemographic characteristics [27,28]. In 2010, NSLP were revised to better reflect the Dietary Guidelines, and increased the amount of low-fat milk, lean meats, seafood, poultry, beans, peas, and unsalted nuts and seeds [29]. Since these changes, one nationally representative study from the USDA reported that NLSP participants had a higher overall diet quality and higher intake of dairy products, but significantly lower seafood and plant protein intake than a set of matched non-participants [30]. Given the lack of data on dietary sources of protein, follow-up data will be helpful in comparing protein food intakes by NSLP/SBP participation status. Our study provides more detailed baseline data than previous studies on potential dietary sources of protein among food assistance participants.

When we examined trends by population subgroups, we found socioeconomic differences. Fish and nuts and seeds intake declined among lower-income children whereas it increased in higher-income children. Further, children from a smaller household size (<3 people) reported a decline in poultry intake. Studies have shown that family size is a determinant of children’s dietary intakes, and nutrient intake is poorer for children living in a single-parent household [31,32]. Our results are in line with previous studies reporting poorer dietary intakes (i.e., lower intakes of lean meats, fish, low-fat dairy, and higher intakes of refined grains and added sugars) among individuals of lower socioeconomic status [33]. These findings underscore the need for health promotion programs for low-income families with children, taking into account greater barriers related to recommended types of protein consumption (higher price of healthier foods, limited access, and lack of knowledge about healthier foods) [13]. Community programs and policies that address multiple barriers may hold a promise for positively influencing children’s dietary intakes [34].

From our data, it appears that adolescents have a different dietary pattern from children and adults in protein food intake [17]. Unlike other age groups, adolescents in higher-income households had a decline in dairy and egg intake, and protein food intake did not differ by household size. Such distinct trends may be considered in light of the fact that in adolescence, there may be greater peer influences, and increased independence with respect to food choices. Further, reports have shown that adolescents consume greater amount of foods away from home, and have weaker resemblance of dietary intakes with their parents than younger children [35,36]. These findings suggest that efforts to increase recommended types of protein should consider different types of intervention strategies for adolescents, recognizing challenges associated with peer influences, eating patterns and emerging independence.

No substantial differences were observed when we adjusted for total energy intake, instead of body weight. However, we found that a decline in beef intake among adolescents was attenuated. Adjusting for body weight does not control for physical activity, which is a greater source of between-person variation in total energy intake than body size [37]. Thus, the attenuation appears to suggest that adolescents who are physically active may not have reduced beef intake over time. We also cannot rule out that the observed attenuation may be due to chance, considering that we conducted a large number of statistical tests. Our findings were relatively robust to adjustment of body size or total energy intake, but we present the results adjusted for body weight to be consistent with how protein is reported in the Dietary References Intakes and to enable comparisons.

To our knowledge, this is the first study which examined the changes in protein food intake in a nationally representative sample of children and adolescents and across a wide range of subgroups over a 10 year period. However, there were several limitations to this study. Although NHANES used standardized approaches to collect dietary data, there are known limitations in assessing intakes of children and adolescents [38,39]. A single 24-hour dietary recall was used to quantify trends in the intake of protein foods, which can describe population-level intake reasonably well, and previous studies used a single 24 hour dietary recall to delineate trends in dietary intakes [13,40–43]. Nevertheless, we observed attenuation in trends of several protein foods when we restricted our analyses to NHANES 2003 to 2010 when two days of dietary recalls were available. This attenuation appears to be due to using only 4 survey cycles, because the two-day averaged intake did not substantially differ from the estimates from a single 24-hour dietary recall, which suggests that one dietary recall can adequately capture population-level intake. In addition, protein food intake could differ by geographical region, but we did not have access to geocoded data. We also could not investigate how trends in subclass of dairy or processed meats has changed over time, because FCID did not have detailed data on low-fat or non-fat yogurt, cheese, milk, or processed meats [16]. Future studies that examine trends in subclass of dairy and processed meats are warranted.

## Conclusions

In conclusion, among US children and adolescents, we found that beef or pork intake declined while chicken and legumes increased over time. However, fish intake did not change, and trends in intake suggested socioeconomic disparities among children. WIC Participants showed favorable trends on recommended types of protein foods compared to income-eligible non-participants. Future research identifying programs and policies to increase recommended types of protein consumption are needed.

## Supporting information

S1 Table Mean intake of different types of protein in US children, stratified by age, National Health and Nutrition Examination Survey 1999–2010. (DOCX)

S2 Table Proportion of US children consuming different types of protein on a given day and mean intake of types of protein among consumers only, stratified by age, National Health and Nutrition Examination Survey 1999–2010. (DOCX)

S3 Table Mean intake of different types of protein in US adolescents and children (2–19 years), stratified by sex, National Health and Nutrition Examination Survey 1999–2010. (DOCX)

S4 Table Mean intake of different types of protein in US children and adolescents (2–19 years), stratified by race/ethnicity, National Health and Nutrition Examination Survey 1999–2010. (DOCX)

S5 Table Mean intake of different types of protein in US children and adolescents (2–19 years), stratified by income, National Health and Nutrition Examination Survey 1999–2010. (DOCX)

S6 Table Mean intake of different types of protein in US children and adolescents (2–19 years), stratified by number of people in a household, National Health and Nutrition Examination Survey 1999–2010. (DOCX)

S7 Table Mean intake of different types of protein in US children and adolescents (2–19 years), stratified by SNAP participation, National Health and Nutrition Examination Survey 1999–2010. (DOCX)

S8 Table Mean intake of different types of protein in US children and adolescents (5-<19 years), stratified by NSLP and SBP participation, National Health and Nutrition Examination Survey 1999–2010. (DOCX)

S9 Table Mean intake of different types of protein in US among children and adolescents, National Health and Nutrition Examination Survey 1999–2010, adjusting for total energy intake instead of body weight. (DOCX)

S10 Table Mean intake of types of protein in US adolescents and children for individuals with two days of dietary recalls. (DOCX)
