# Supplementary material for: Trends in types of protein in US adolescents and children: Results from the National Health and Nutrition Examination Survey 1999-2010
Source: PLoS One. 2020 Mar 26;15(3):e0230686. doi: 10.1371/journal.pone.0230686 (PMC7098572; doi:10.1371/journal.pone.0230686)
Supplement: S1 Table — (DOCX) [file pone.0230686.s001.docx]

S1 Table. Mean intake of different types of protein in US children, stratified by age, National Health and Nutrition Examination Survey 1999-2010

|  | 1999-2000 | 2001-2002 | 2003-2004 | 2005-2006 | 2007-2008 | 2009-2010 |  |  |
| --- | --- | --- | --- | --- | --- | --- | --- | --- |
|  | (n=1,701) | (n=1,995) | (n=1,665) | (n=1,917) | (n=1,953) | (n=2,020) | Percent change^2^ |  |
| Intake in grams of protein foods (g) per kg of body weight ± SE for the overall study population^1^ | | | | | | | | *P-*trend |
|  | 2-<6 years of age | | | | | |  |  |
| Sample size | 713 | 859 | 765 | 905 | 832 | 866 |  |  |
| Beef | 1.66 ± 0.18 | 1.46 ± 0.1 | 1.61 ± 0.16 | 1.31 ± 0.11 | 1.38 ± 0.09 | 1.33 ± 0.09 | -19.9 | 0.046 |
| Pork | 0.74 ± 0.11 | 0.54 ± 0.06 | 0.75 ± 0.06 | 0.84 ± 0.09 | 0.72 ± 0.05 | 0.66 ± 0.07 | -10.8 | 0.77 |
| Lamb or goat | 0.01 ± 0.01 | 0.02 ± 0.01 | 0.02 ± .01 | 0.02 ± 0.01 | 0.01 ± .05 | 0.01 ± 0.004 | 0 | 0.39 |
| Chicken | 1.28 ± 0.09 | 0.95 ± 0.13 | 1.48 ± 0.09 | 1.36 ± 0.09 | 1.53 ± 0.10 | 1.62 ± 0.004 | 26.6 | <0.001 |
| Turkey | 0.24 ± 0.04 | 0.29 ± 0.07 | 0.32 ± 0.06 | 0.24 ± 0.03 | 0.35 ± 0.03 | 0.34 ± 0.04 | 41.7 | 0.14 |
| All poultry | 1.52 ± 0.11 | 1.23 ± 0.18 | 1.8 ± 0.10 | 1.6 ± 0.11 | 1.88 ± 0.12 | 1.96 ± 0.12 | 28.9 | <0.001 |
| Fish and shellfish | 0.18 ± 0.04 | 0.35 ± 0.07 | 0.28 ± .08 | 0.34 ± 0.10 | 0.22 ± .04 | 0.25 ± 0.08 | 38.9 | 0.97 |
| Milk and Milk products | 24.82 ± 1.81 | 25.5 ± 1.55 | 27.8 ± 1.23 | 25.22 ± 0.88 | 26.36 ± 1.23 | 27.64 ± 1.09 | 11.4 | 0.23 |
| Eggs | 0.84 ± 0.07 | 0.79 ± 0.09 | 0.89 ± 0.08 | 0.84 ± 0.06 | 1.04 ± 0.09 | 0.89 ± 0.05 | 6.0 | 0.14 |
| Legumes | 0.59 ± 0.08 | 0.5 ± 0.12 | 1.01 ± 0.35 | 1.02 ± 0.24 | 1.01 ± 0.16 | 0.79 ± 0.10 | 33.9 | 0.02 |
| Nuts and Seeds | 0.57 ± 0.07 | 0.44 ± 0.06 | 0.47 ± 0.05 | 0.45 ± 0.05 | 0.35 ± 0.03 | 0.54 ± 0.06 | -5.3 | 0.44 |
|  | 6-<12 years of age | | | | | |  |  |
| Sample Size | 988 | 1,136 | 900 | 1,012 | 1,121 | 1,154 |  |  |
| Beef | 1.20 ± 0.12 | 1.17 ± 0.07 | 1.19 ± 0.06 | 0.92 ± 0.05 | 1.30 ± 0.13 | 1.00 ± 0.07 | -16.7 | 0.31 |
| Pork | 0.76 ± 0.07 | 0.58 ± 0.04 | 0.49 ± 0.05 | 0.56 ± 0.05 | 0.54 ± 0.03 | 0.51 ± 0.03 | -32.9 | 0.005 |
| Lamb or goat | 0.01 ± 0.01 | 0.01 ± 0.01 | 0.02 ± .02 | 0.01 ± .01 | 0.01 ± .01 | 0.01 ± 0.02 | 0 | 0.47 |
| Chicken | 0.78 ± 0.05 | 0.84 ± 0.08 | 0.98 ± 0.11 | 0.95 ± 0.10 | 0.97 ± 0.08 | 1.06 ± 0.07 | 35.9 | 0.002 |
| Turkey | 0.18 ± 0.03 | 0.17 ± 0.02 | 0.23 ± 0.05 | 0.19 ± 0.03 | 0.22 ± 0.04 | 0.20 ± 0.02 | 11.1 | 0.37 |
| All poultry | 0.96 ± 0.07 | 1.01 ± 0.09 | 1.20 ± 0.10 | 1.13 ± 0.11 | 1.19 ± 0.1 | 1.26 ± 0.07 | 31.3 | 0.003 |
| Fish and shellfish | 0.19 ± 0.03 | 0.23 ± 0.07 | 0.13 ± 0.02 | 0.23 ± 0.08 | 0.16 ± 0.02 | 0.18 ± 0.03 | -5.3 | 0.77 |
| Milk and Milk products | 12.38 ± 0.61 | 12.96 ± 0.6 | 13.26 ± 0.78 | 12.88 ± 0.44 | 11.14 ± 0.41 | 12.62 ± 0.41 | 1.9 | 0.20 |
| Eggs | 0.49 ± 0.04 | 0.45 ± 0.05 | 0.55 ± 0.07 | 0.64 ± 0.04 | 0.50 ± 0.04 | 0.55 ± 0.03 | 12.2 | 0.18 |
| Legumes | 0.19 ± 0.04 | 0.25 ± 0.05 | 0.32 ± 0.05 | 0.31 ± 0.06 | 0.41 ± 0.11 | 0.37 ± 0.06 | 94.7 | 0.01 |
| Nuts and Seeds | 0.38 ± 0.08 | 0.3 ± 0.03 | 0.36 ± 0.06 | 0.29 ± 0.02 | 0.27 ± 0.03 | 0.32 ± 0.03 | -15.8 | 0.33 |

^1^ /kg indicates grams of protein food intake per kilogram of body weight, and SE indicates standard errors.

^2^ Percent change from 1999-2000 to 2009-2010
