# Supplementary material for: Trends in types of protein in US adolescents and children: Results from the National Health and Nutrition Examination Survey 1999-2010
Source: PLoS One. 2020 Mar 26;15(3):e0230686. doi: 10.1371/journal.pone.0230686 (PMC7098572; doi:10.1371/journal.pone.0230686)
Supplement: S2 Table — (DOCX) [file pone.0230686.s002.docx]

S2 Table. Proportion of US children consuming different types of protein on a given day and mean intake of types of protein among consumers only, stratified by age, National Health and Nutrition Examination Survey 1999-2010

|  | 1999-2000 | 2001-2002 | 2003-2004 | 2005-2006 | 2007-2008 | 2009-2010 | Percent change^1^ | *P*-trend |
| --- | --- | --- | --- | --- | --- | --- | --- | --- |
|  | 2-<6 years of age: Percent of Consumers, % | | | | | |  |  |
| Beef | 70 | 69 | 79 | 71 | 72 | 70 | 0 | 0.92 |
| Pork | 58 | 55 | 63 | 57 | 60 | 57 | -1.7 | 0.91 |
| Lamb or goat | 5 | 5 | 6 | 6 | 5 | 6 | 20.0 | 0.88 |
| Chicken | 49 | 39 | 55 | 54 | 58 | 58 | 18.4 | <0.001 |
| Turkey | 21 | 19 | 26 | 22 | 30 | 26 | 23.8 | 0.003 |
| All poultry | 55 | 44 | 61 | 56 | 61 | 62 | 12.7 | 0.001 |
| Fish and shellfish | 8 | 10 | 10 | 12 | 8 | 10 | 25.0 | 0.84 |
| Milk and Milk products | 99 | 89 | 100 | 100 | 100 | 100 | 1.0 | <0.001 |
| Eggs | 82 | 73 | 85 | 83 | 85 | 82 | 0 | 0.11 |
| Legumes | 72 | 65 | 74 | 71 | 71 | 65 | -9.7 | 0.32 |
| Nuts and Seeds | 78 | 70 | 80 | 76 | 76 | 78 | 0 | 0.20 |
| 2-<6 years of age: Intake in grams of protein foods (g) per kg of body weight ± SE among consumers only^3^ | | | | | | | | |
| Beef | 2.38 ± 0.17 | 2.12 ± 0.12 | 2.05 ± 0.20 | 1.83 ± 0.15 | 1.91 ± 0.12 | 1.90 ± 0.11 | -20.2 | 0.006 |
| Pork | 1.29 ± 0.13 | 0.99 ± 0.08 | 1.2 0± 0.08 | 1.47 ± 0.13 | 1.19 ± 0.09 | 1.15 ± 0.11 | -10.9 | 0.76 |
| Lamb or goat | 0.28 ± 0.16 | 0.39 ± 0.25 | 0.33 ± 0.22 | 0.31 ± 0.16 | 0.19 ± 0.18 | 0.18 ± 0.08 | -35.7 | 0.37 |
| Chicken | 2.60 ± 0.11 | 2.45 ± 0.22 | 2.71 ± 0.15 | 2.54 ± 0.13 | 2.64 ± 0.08 | 2.78 ± 0.15 | 6.9 | 0.28 |
| Turkey | 1.13 ± 0.16 | 1.53 ± 0.20 | 1.21 ± 0.20 | 1.11 ± 0.16 | 1.18 ± 0.10 | 1.28 ± 0.16 | 13.3 | 0.75 |
| All poultry | 2.79 ± 0.12 | 2.84 ± 0.22 | 2.98 ± 0.18 | 2.88 ± 0.17 | 3.07 ± 0.11 | 3.16 ± 0.14 | 13.3 | 0.04 |
| Fish and shellfish | 2.26 ± 0.43 | 3.43 ± 0.51 | 2.88 ± 0.42 | 3.00 ± 0.59 | 2.75 ± 0.23 | 2.6 ± 0.41 | 15.0 | 0.75 |
| Milk and Milk products | 25.06 ± 1.79 | 28.79 ± 1.44 | 27.84 ± 1.24 | 25.24 ± 0.88 | 26.4 ± 1.22 | 27.75 ± 1.10 | 10.7 | 0.76 |
| Eggs | 1.02 ± 0.09 | 1.08 ± 0.11 | 1.04 ± 0.10 | 1.02 ± 0.09 | 1.23 ± 0.12 | 1.09 ± 0.07 | 6.9 | 0.38 |
| Legumes | 0.82 ± 0.13 | 0.77 ± 0.19 | 1.37 ± 0.48 | 1.43 ± 0.31 | 1.42 ± 0.22 | 1.20 ± 0.14 | 46.3 | 0.009 |
| Nuts and Seeds | 0.73 ± 0.10 | 0.63 ± 0.07 | 0.59 ± 0.06 | 0.58 ± 0.06 | 0.47 ± 0.04 | 0.69 ± 0.08 | -5.5 | 0.29 |
| 6-<12 years of age: Percent of Consumers, %^2^ | | | | | | | | |
| Beef | 76 | 78 | 78 | 69 | 79 | 74 | -2.6 | 0.41 |
| Pork | 66 | 66 | 65 | 63 | 68 | 61 | -7.6 | 0.34 |
| Lamb or goat | 4 | 8 | 8 | 5 | 7 | 5 | 25.0 | 0.57 |
| Chicken | 45 | 46 | 49 | 52 | 50 | 51 | 13.3 | 0.02 |
| Turkey | 24 | 24 | 22 | 22 | 26 | 26 | 8.3 | 0.54 |
| All poultry | 50 | 50 | 52 | 56 | 54 | 56 | 12.0 | 0.06 |
| Fish and shellfish | 10 | 11 | 10 | 13 | 9 | 11 | 10.0 | 0.6 |
| Milk and Milk products | 99 | 98 | 100 | 100 | 100 | 100 | 1.0 | <0.001 |
| Eggs | 81 | 83 | 86 | 87 | 86 | 82 | 1.2 | 0.41 |
| Legumes | 68 | 67 | 73 | 67 | 68 | 69 | 1.5 | 0.73 |
| Nuts and Seeds | 79 | 75 | 78 | 80 | 80 | 79 | 0 | 0.31 |
| 6-<12 years of age: Intake in grams of protein foods (g) per kg of body weight ± SE among consumers only^2^ | | | | | | | | |
| Beef | 1.59 ± 0.16 | 1.50 ± 0.08 | 1.53 ± 0.07 | 1.34 ± 0.06 | 1.65 ± 0.15 | 1.36 ± 0.07 | -14.5 | 0.45 |
| Pork | 1.15 ± 0.10 | 0.88 ± 0.05 | 0.77 ± 0.08 | 0.88 ± 0.07 | 0.80 ± 0.04 | 0.83 ± 0.05 | -27.8 | 0.01 |
| Lamb or goat | 0.16 ± 0.11 | 0.08 ± 0.05 | 0.29 ± 0.22 | 0.29 ± 0.13 | 0.19 ± 0.11 | 0.30 ± 0.19 | 87.5 | 0.34 |
| Chicken | 1.76 ± 0.09 | 1.84 ± 0.13 | 2.00 ± 0.18 | 1.81 ± 0.11 | 1.94 ± 0.13 | 2.07 ± 0.11 | 17.6 | 0.06 |
| Turkey | 0.75 ± 0.05 | 0.70 ± 0.07 | 1.04 ± 0.14 | 0.85 ± 0.09 | 0.86 ± 0.10 | 0.76 ± 0.05 | 1.3 | 0.57 |
| All poultry | 1.92 ± 0.08 | 2.03 ± 0.14 | 2.34 ± 0.13 | 2.02 ± 0.13 | 2.20 ± 0.15 | 2.26 ± 0.09 | 17.7 | 0.03 |
| Fish and shellfish | 1.94 ± 0.21 | 2.17 ± 0.36 | 1.38 ± 0.20 | 1.72 ± 0.20 | 1.80 ± 0.16 | 1.68 ± 0.16 | -13.4 | 0.28 |
| Milk and Milk products | 12.46 ± 0.61 | 13.23 ± 0.64 | 13.32 ± 0.79 | 12.91 ± 0.44 | 11.15 ± 0.42 | 12.63 ± 0.41 | 1.4 | 0.13 |
| Eggs | 0.61 ± 0.05 | 0.55 ± 0.05 | 0.64 ± 0.08 | 0.73 ± 0.05 | 0.58 ± 0.05 | 0.67 ± 0.03 | 9.8 | 0.24 |
| Legumes | 0.29 ± 0.05 | 0.38 ± 0.07 | 0.44 ± 0.08 | 0.46 ± 0.08 | 0.60 ± 0.16 | 0.55 ± 0.09 | 89.7 | 0.009 |
| Nuts and Seeds | 0.48 ± 0.09 | 0.39 ± 0.04 | 0.46 ± 0.07 | 0.37 ± 0.02 | 0.34 ± 0.04 | 0.41 ± 0.04 | -14.6 | 0.25 |

^1^ Consumers are defined as those who consumed a specific type of protein more than 0 grams a day.

^2^ Percent change from 1999-2000 to 2009-2010

^3^ g/kg indicates grams of protein food intake per kilogram of body weight, and SE indicates standard errors.
