# Supplementary material for: Trends in types of protein in US adolescents and children: Results from the National Health and Nutrition Examination Survey 1999-2010
Source: PLoS One. 2020 Mar 26;15(3):e0230686. doi: 10.1371/journal.pone.0230686 (PMC7098572; doi:10.1371/journal.pone.0230686)
Supplement: S3 Table — (DOCX) [file pone.0230686.s003.docx]

S3 Table. Mean intake of different types of protein in US adolescents and children (2-19 years), stratified by sex, National Health and Nutrition Examination Survey 1999-2010

|  | Boys | |  | Girls | |  |  |
| --- | --- | --- | --- | --- | --- | --- | --- |
| Intake in grams of protein foods (g) per kg of body weight ± SE^1^ | | | | | | | |
|  | 1999-2000 | 2009-2010 | Percent change^2^ | 1999-2000 | 2009-2010 | Percent change^2^ |  |
|  | (n=872) | (n=1,042) |  | (n=829) | (n=978) |  |  |
|  | Children (2-<12 years of age) | | | | | | *P*-interaction |
| Beef | 1.43 ±0.14 | 1.17 ±0.06^*^ | -18.2 | 1.33 ±0.16 | 1.09 ±0.10 | -18.0 | 0.71 |
| Pork | 0.84 ±0.12 | 0.6 ±0.05 | -28.6 | 0.66 ±0.05 | 0.54 ±0.05 | -18.2 | 0.80 |
| Lamb or goat | 0.01 ±0.01 | 0.02 ±0.01 | 100.0 | 0.01 ±0.01 | 0.01 ±0.01 | 0 | 0.40 |
| Chicken | 0.95 ±0.08 | 1.29 ±0.09^***^ | 35.8 | 1.01 ±0.08 | 1.29 ±0.08^*^ | 27.7 | 0.31 |
| Turkey | 0.20 ±0.03 | 0.24 ±0.04 | 20.0 | 0.2 ±0.04 | 0.27 ±0.02 | 35.0 | 0.85 |
| All Poultry | 1.15 ±0.09 | 1.52 ±0.11^***^ | 32.2 | 1.21 ±0.1 | 1.56 ±0.1^**^ | 30.0 | 0.39 |
| Fish and shellfish | 0.21 ±0.05 | 0.18 ±0.03 | -14.3 | 0.16 ±0.04 | 0.24 ±0.07 | 50.0 | 0.04 |
| Milk and Milk products | 18.41 ±1.47 | 19.09 ±0.51 | 3.7 | 16.0 ±0.75 | 18.23 ±0.92 | 13.9 | 0.88 |
| Eggs | 0.71 ±0.04 | 0.69 ±0.06^*^ | -2.8 | 0.54 ±0.05 | 0.68 ±0.05^*^ | 25.9 | 0.05 |
| Legumes | 0.32 ±0.05 | 0.48 ±0.13^*^ | 50.0 | 0.37 ±0.06 | 0.61 ±0.11 | 64.9 | 0.49 |
| Nuts and Seeds | 0.51 ±0.08 | 0.44 ±0.03 | -13.7 | 0.39 ±0.05 | 0.37 ±0.04 | -5.1 | 0.19 |
| Adolescents (12-19 years of age) | | | | | | | |
|  | 1999-2000 | 2009-2010 |  | 1999-2000 | 2009-2010 |  |  |
|  | (n=1,113) | (n=672) |  | (n=1,106) | (n=593) |  |  |
| Beef | 1.19 ± 0.12 | 0.88 ± 0.09^*^ | -26.1 | 0.63 ± 0.08 | 0.48 ± 0.04^*^ | -23.8 | 0.81 |
| Pork | 0.43 ± 0.04 | 0.53 ± 0.08 | 23.3 | 0.26 ± 0.03 | 0.29 ± 0.03 | 11.5 | 0.23 |
| Lamb or goat | 0.02 ± 0.01 | 0.01 ± 0.01 | -50.0 | 0.01 ± 0.01 | 0.03 ± 0.02 | 200.0 | 0.50 |
| Chicken | 0.60 ± 0.07 | 0.82 ± 0.05^**^ | 36.7 | 0.59 ± 0.06 | 0.66 ± 0.07^*^ | 11.9 | 0.02 |
| Turkey | 0.13 ± 0.02 | 0.13 ± 0.01 | 0 | 0.12 ± 0.02 | 0.12 ± 0.01 | 0 | 0.26 |
| All Poultry | 0.73 ± 0.07 | 0.95 ± 0.05^*^ | 30.1 | 0.71 ± 0.07 | 0.78 ± 0.07^**^ | 9.9 | 0.02 |
| Fish and shellfish | 0.10 ± 0.03 | 0.13 ± 0.03 | 30.0 | 0.13 ± 0.02 | 0.11 ± 0.02 | -15.4 | 0.65 |
| Milk and Milk products | 6.5 ± 0.44 | 6.03 ± 0.71 | -7.2 | 4.75 ± 0.35 | 4.65 ± 0.35 | -2.1 | 0.68 |
| Eggs | 0.32 ± 0.03 | 0.30 ± 0.04 | -6.3 | 0.24 ± 0.03 | 0.35 ± 0.03^*^ | 45.8 | 0.03 |
| Legumes | 0.13 ± 0.03 | 0.13 ± 0.03 | 0 | 0.14 ± 0.05 | 0.18 ± 0.04 | 28.6 | 0.87 |
| Nuts and Seeds | 0.18 ± 0.03 | 0.22 ± 0.03 | 22.2 | 0.15 ± 0.01 | 0.21 ± 0.03 | 40.0 | 0.80 |

^1^ Linearized standard error

^2^ Percent change from 1999-2000 to 2009-2010
