# Supplementary material for: Trends in types of protein in US adolescents and children: Results from the National Health and Nutrition Examination Survey 1999-2010
Source: PLoS One. 2020 Mar 26;15(3):e0230686. doi: 10.1371/journal.pone.0230686 (PMC7098572; doi:10.1371/journal.pone.0230686)
Supplement: S4 Table — (DOCX) [file pone.0230686.s004.docx]

S4A Table. Mean intake of different types of protein in US children and adolescents (2-19 years), stratified by race/ethnicity, National Health and Nutrition Examination Survey 1999-2010

|  | non-Hispanic white | | | non-Hispanic black | | |
| --- | --- | --- | --- | --- | --- | --- |
|  | Intake in grams of protein foods (g) per kg of body weight ± SE^1^ | | | | | |
|  | 1999-2000 | 2009-2010 | Percent change^2^ | 1999-2000 | 2009-2010 | Percent change^2^ |
|  | (n=408) | (n=678) |  | (n=480) | (n=379) |  |
|  | Children (2-<12 years of age) | | | | | |
| Beef | 1.38 ± 0.17 | 1.10 ± 0.11 | -20.3 | 1.43 ± 0.12 | 1.36 ± 0.14 | -4.9 |
| Pork | 0.73 ± 0.12 | 0.56 ± 0.07 | -23.3 | 0.76 ± 0.08 | 0.56 ± 0.11 | -26.3 |
| Lamb or goat | 0.01 ± 0.01 | 0.02 ± 0.01 | 100.0 | 0.02 ± 0.02 | 0.02 ± 0.01 | 0 |
| Chicken | 0.87 ± 0.08 | 1.04 ± 0.08^*^ | 19.5 | 1.32 ± 0.14 | 1.82 ± 0.09^**^ | 37.9 |
| Turkey | 0.20 ± 0.04 | 0.24 ± 0.02^*^ | 20.0 | 0.30 ± 0.06 | 0.24 ± 0.04^**^ | -20.0 |
| All Poultry | 1.07 ± 0.10 | 1.28 ± 0.09^*^ | 19.6 | 1.62 ± 0.16 | 2.06 ± 0.09^*^ | 27.2 |
| Fish and shellfish | 0.19 ± 0.04 | 0.21 ± 0.08 | 10.5 | 0.22 ± 0.05 | 0.18 ± 0.04 | -18.2 |
| Milk and Milk products | 17.23 ± 1.19 | 20.29 ± 0.95 | 17.8 | 12.62 ± 0.56 | 13.5 ± 1.27 | 7.0 |
| Eggs | 0.52 ± 0.06 | 0.59 ± 0.06 | 13.5 | 0.59 ± 0.02 | 0.60 ± 0.07 | 1.7 |
| Legumes | 0.34 ± 0.06 | 0.66 ± 0.13^**^ | 94.1 | 0.52 ± 0.06 | 0.41 ± 0.08 | -21.2 |
| Nuts and Seeds | 0.50 ± 0.07 | 0.41 ± 0.04 | -18.0 | 0.28 ± 0.04 | 0.37 ± 0.07 | 32.1 |
|  | Adolescents (12-19 years of age) | | | | | |
|  | 1999-2000 | 2009-2010 |  | 1999-2000 | 2009-2010 | Percent change^2^ |
|  | (n=446) | (n=425) |  | (n=612) | (n=275) |  |
| Beef | 0.92 ±0.12 | 0.69 ±0.07 | -25 | 0.75 ±0.04 | 0.65 ±0.05^*^ | -13.3 |
| Pork | 0.32 ±0.04 | 0.37 ±0.06 | 15.6 | 0.45 ±0.05 | 0.26 ±0.03^**^ | -42.2 |
| Lamb or goat | 0.01 ±0.01 | 0.03 ±0.02 | 200.0 | 0.02 ±0.02 | 0.01 ±0.01 | -50.0 |
| Chicken | 0.54 ±0.06 | 0.67 ±0.07^*^ | 24.1 | 0.76 ±0.06 | 0.86 ±0.09 | 13.2 |
| Turkey | 0.11 ±0.02 | 0.13 ±0.01 | 18.2 | 0.13 ±0.03 | 0.11 ±0.02 | -15.4 |
| All Poultry | 0.65 ±0.06 | 0.80 ±0.06^**^ | 23.1 | 0.89 ±0.07 | 0.97 ±0.10 | 9.0 |
| Fish and shellfish | 0.08 ±0.02 | 0.11 ±0.03 | 37.5 | 0.16 ±0.05 | 0.10 ±0.03 | -37.5 |
| Milk and Milk products | 6.63 ±0.42 | 5.99 ±0.69 | -9.7 | 3.79 ±0.32 | 3.39 ±0.3 | -12.9 |
| Eggs | 0.26 ±0.02 | 0.28 ±0.04 | 7.7 | 0.32 ±0.03 | 0.36 ±0.11 | 12.5 |
| Legumes | 0.11 ±0.03 | 0.16 ±0.04 | 45.5 | 0.15 ±0.02 | 0.15 ±0.05 | 0 |
| Nuts and Seeds | 0.14 ±0.02 | 0.2 ±0.04 | 42.9 | 0.14 ±0.02 | 0.18 ±0.06 | 28.6 |

S4B Table. Mean intake of different types of protein in US children and adolescents (2-19 years), stratified by race/ethnicity, National Health and Nutrition Examination Survey 1999-2010

|  | Mexican American | | | other | | |  |
| --- | --- | --- | --- | --- | --- | --- | --- |
|  | | | | | | | |
|  | 1999-2000 | 2009-2010 | Percent change^2^ | 1999-2000 | 2009-2010 | Percent change^2^ |  |
|  | (n=748) | (n=809) |  | (n=65) | (n=154) |  |  |
|  | Children (2-<12 years of age) | | | | | | *P*-interaction |
| Beef | 1.34 ± 0.09 | 1.15 ± 0.10 | -14.2 | 1.44 ± 0.37 | 0.90 ± 0.18^*^ | -37.5 | 0.51 |
| Pork | 0.84 ± 0.08 | 0.58 ± 0.07 | -31.0 | 0.65 ± 0.28 | 0.65 ± 0.07 | 0 | 0.88 |
| Lamb or goat | 0.01 ± 0.01 | 0.01 ± 0.01 | 0 | 0.01 ± 0.01 | 0.01 ± 0.01 | 0 | 0.10 |
| Chicken | 0.96 ± 0.1 | 1.54 ± 0.11^***^ | 60.4 | 1.32 ± 0.41 | 1.43 ± 0.2 | 8.3 | 0.10 |
| Turkey | 0.16 ± 0.03 | 0.21 ± 0.04^***^ | 31.3 | 0.06 ± 0.03 | 0.45 ± 0.23 | 650.0 | 0.34 |
| All Poultry | 1.13 ± 0.11 | 1.75 ± 0.12^*^ | 54.9 | 1.39 ± 0.41 | 1.89 ± 0.26 | 36.0 | 0.04 |
| Fish and shellfish | 0.14 ± 0.05 | 0.22 ± 0.03 | 57.1 | 0.14 ± 0.03 | 0.29 ± 0.10 | 107.1 | 0.36 |
| Milk and Milk products | 19.99 ± 1.58 | 17.84 ± 0.77 | -10.8 | 19.8 ± 3.07 | 18.6 ± 1.73 | -6.1 | 0.11 |
| Eggs | 1.04 ± 0.11 | 0.92 ± 0.13 | -11.5 | 0.48 ± 0.1 | 0.81 ± 0.11 | 68.8 | 0.48 |
| Legumes | 0.21 ± 0.04 | 0.23 ± 0.06 | 9.5 | 0.45 ± 0.24 | 0.81 ± 0.23 | 80.0 | 0.20 |
| Nuts and Seeds | 0.47 ± 0.09 | 0.42 ± 0.04 | -10.6 | 0.36 ± 0.12 | 0.46 ± 0.12 | 27.8 | 0.05 |
| Adolescents (12-19 years of age) | | | | | | | |
|  | 1999-2000 | 2009-2010 | Percent change^2^ | 1999-2000 | 2009-2010 | Percent change^2^ |  |
|  | (n=748) | (n=809) |  | (n=65) | (n=154) |  | *P*-interaction |
| Beef | 0.89 ±0.08 | 0.68 ±0.05^**^ | -23.6 | 1.13 ±0.26 | 0.56 ±0.13 | -50.4 | 0.57 |
| Pork | 0.35 ±0.02 | 0.39 ±0.05 | 11.4 | 0.34 ±0.07 | 1.08 ±0.47 | 217.6 | 0.11 |
| Lamb or goat | 0.01 ±0.01 | 0.01 ±0.01 | 0 | 0.01 ±0.01 | 0.01 ±0.01 | 0 | 0.19 |
| Chicken | 0.55 ±0.03 | 0.91 ±0.09^**^ | 65.5 | 0.75 ±0.22 | 0.58 ±0.12 | -22.7 | 0.15 |
| Turkey | 0.10 ±0.02 | 0.14 ±0.03 | 40.0 | 0.25 ±0.12 | 0.07 ±0.02 | -72.0 | 0.37 |
| All Poultry | 0.65 ±0.05 | 1.04 ±0.10^***^ | 60.0 | 1.00 ±0.28 | 0.65 ±0.13 | -35.0 | 0.17 |
| Fish and shellfish | 0.12 ±0.02 | 0.09 ±0.02 | -25.0 | 0.22 ±0.12 | 0.31 ±0.1 | 36.4 | 0.006 |
| Milk and Milk products | 5.36 ±0.22 | 4.48 ±0.38^*^ | -16.4 | 3.10 ±0.45 | 5.75 ±0.85 | 85.5 | 0.21 |
| Eggs | 0.36 ±0.02 | 0.37 ±0.05 | 2.8 | 0.23 ±0.06 | 0.48 ±0.11^*^ | 108.7 | 0.20 |
| Legumes | 0.07 ±0.03 | 0.12 ±0.04 | 71.4 | 0.33 ±0.21 | 0.27 ±0.17 | -18.2 | 0.85 |
| Nuts and Seeds | 0.25 ±0.03 | 0.28 ±0.06 | 12.0 | 0.2 ±0.09 | 0.21 ±0.07 | 5.0 | 0.06 |
