# Supplementary material for: Trends in types of protein in US adolescents and children: Results from the National Health and Nutrition Examination Survey 1999-2010
Source: PLoS One. 2020 Mar 26;15(3):e0230686. doi: 10.1371/journal.pone.0230686 (PMC7098572; doi:10.1371/journal.pone.0230686)
Supplement: S5 Table — (DOCX) [file pone.0230686.s005.docx]

S5 Table. Mean intake of different types of protein in US children and adolescents (2-19 years), stratified by income, National Health and Nutrition Examination Survey 1999-2010

|  | | ≤1.30^1^ | | | | | 1.31-<3.50 | | | ≥3.50 | | | |  |
| --- | --- | --- | --- | --- | --- | --- | --- | --- | --- | --- | --- | --- | --- | --- |
|  | Intake in grams of protein foods (g) per kg of body weight ± SE^2^ | | | | | | | | | | | | | |
|  | | 1999-2000 | | 2009-2010 | Percent change^3^ | | 1999-2000 | 2009-2010 | Percent change^3^ | 1999-2000 | 2009-2010 | Percent change^3^ | |  |
|  | | (n=694) | | (n=929) |  | | (n=515) | (n=604) |  | (n=246) | (n=325) |  | |  |
| Children (2-<12 years of age) | | | | | | | | | | | | | *P*-interaction | |
| Beef | | 1.43 ± 0.22 | 1.36 ± 0.13 | | | -4.9 | 1.26 ± 0.12 | 1.08 ± 0.07 | -14.3 | 1.33 ± 0.21 | 0.93 ± 0.13 | -30.1 | | 0.63 |
| Pork | | 0.88 ± 0.10 | 0.65 ± 0.06 | | | -26.1 | 0.64 ± 0.09 | 0.51 ± 0.06 | -20.3 | 0.64 ± 0.11 | 0.54 ± 0.09 | -15.6 | | 0.61 |
| Lamb or goat | | 0.02 ± 0.003 | 0.01 ± 0.01 | | | -50.0 | 0.01 ± 0.01 | 0.03 ± 0.02 | 200.0 | 0.01 ± 0.01 | 0.01 ± 0.01 | 0 | | 0.36 |
| Chicken | | 0.98 ± 0.12 | 1.60 ± 0.13^***^ | | | 63.3 | 0.95 ± 0.07 | 1.10 ± 0.12 | 15.8 | 1.00 ± 0.14 | 1.09 ± 0.13 | 9.0 | | 0.69 |
| Turkey | | 0.23 ± 0.04 | 0.33 ± 0.04^*^ | | | 43.5 | 0.16 ± 0.03 | 0.21 ± 0.02 | 31.3 | 0.18 ± 0.06 | 0.22 ± 0.04 | 22.2 | | 0.43 |
| All Poultry | | 1.21 ± 0.14 | 1.93 ± 0.14^***^ | | | 59.5 | 1.12 ± 0.09 | 1.31 ± 0.12 | 17.0 | 1.18 ± 0.15 | 1.31 ± 0.16 | 11.0 | | 0.56 |
| Fish and shellfish | | 0.27 ± 0.09 | 0.19 ± 0.04 | | | -29.6 | 0.13 ± 0.04 | 0.12 ± 0.03 | -7.7 | 0.11 ± 0.03 | 0.26 ± 0.05 | 136.4 | | 0.09 |
| Milk and Milk products | | 16.94 ± 1.23 | 18.69 ± 1.03 | | | 10.3 | 17.51 ± 1.18 | 20.68 ± 1.11 | 18.1 | 17.25 ± 2.30 | 16.50 ± 1.40 | -4.3 | | 0.23 |
| Eggs | | 0.82 ± 0.08 | 0.77 ± 0.07 | | | -6.1 | 0.51 ± 0.08 | 0.62 ± 0.04^*^ | 21.6 | 0.49 ± 0.09 | 0.59 ± 0.07 | 20.4 | | 0.91 |
| Legumes | | 0.39 ± 0.07 | 0.45 ± 0.07 | | | 15.4 | 0.24 ± 0.05 | 0.48 ± 0.12^*^ | 100 | 0.40 ± 0.08 | 0.76 ± 0.26^*^ | 90.0 | | 0.32 |
| Nuts and Seeds | | 0.37 ± 0.07 | 0.39 ± 0.03 | | | 5.4 | 0.53 ± 0.13 | 0.38 ± 0.05 | -28.3 | 0.36 ± 0.04 | 0.47 ± 0.09 | 30.6 | | 0.07 |
| Adolescents (12-19 years of age) | | | | | | | | | | | | | | |
|  | | 1999-2000 | | 2009-2010 | | Percent change^3^ | 1999-2000 | 2009-2010 | Percent change^3^ | 1999-2000 | 2009-2010 | Percent change^3^ | |  |
|  | | (n=934) | | (n=503) | |  | (n=623) | (n=413) |  | (n=360) | (n=231) |  | |  |
| Beef | | 0.94 ± 0.17 | | 0.68 ± 0.06 | | -27.7 | 0.86 ± 0.08 | 0.60 ± 0.04 | -30.2 | 0.83 ± 0.10 | 0.77 ± 0.15 | -7.2 | | 0.28 |
| Pork | | 0.36 ± 0.04 | | 0.51 ± 0.12 | | 41.7 | 0.37 ± 0.05 | 0.37 ± 0.08 | 0 | 0.31 ± 0.04 | 0.42 ± 0.09 | 35.5 | | 0.77 |
| Lamb or goat | | 0.01 ± 0.01 | | 0.01 ± 0.01 | | 0 | 0.02 ± 0.02 | 0.01 ± 0.01 | -50.0 | 0.01 ± 0.01 | 0.04 ± 0.04 | 300.0 | | 0.47 |
| Chicken | | 0.66 ± 0.09 | | 0.71 ± 0.10^**^ | | 7.6 | 0.50 ± 0.06 | 0.74 ± 0.06^**^ | 48.0 | 0.61 ± 0.08 | 0.71 ± 0.12 | 16.4 | | 0.78 |
| Turkey | | 0.13 ± 0.03 | | 0.12 ± 0.02 | | -7.7 | 0.09 ± 0.02 | 0.09 ± 0.02 | 0 | 0.11 ± 0.01 | 0.15 ± 0.03 | 36.4 | | 0.44 |
| All Poultry | | 0.79 ± 0.12 | | 0.84 ± 0.11 | | 6.3 | 0.59 ± 0.06 | 0.83 ± 0.07^**^ | 40.7 | 0.72 ± 0.08 | 0.87 ± 0.13 | 20.8 | | 0.91 |
| Fish and shellfish | | 0.15 ± 0.04 | | 0.09 ± 0.03 | | -40.0 | 0.10 ± 0.04 | 0.13 ± 0.04 | 30.0 | 0.08 ± 0.02 | 0.14 ± 0.03 | 75.0 | | 0.49 |
| Milk and Milk products | | 4.89 ± 0.37 | | 5.21 ± 0.46 | | 6.5 | 6.38 ± 0.51 | 4.90 ± 0.64^**^ | -23.2 | 5.76 ± 0.54 | 5.52 ± 0.47 | -4.2 | | 0.04 |
| Eggs | | 0.26 ± 0.02 | | 0.33 ± 0.06 | | 26.9 | 0.25 ± 0.03 | 0.32 ± 0.04 | 28.0 | 0.31 ± 0.04 | 0.26 ± 0.04 | -16.1 | | 0.05 |
| Legumes | | 0.15 ± 0.03 | | 0.17 ± 0.05 | | 13.3 | 0.13 ± 0.05 | 0.14 ± 0.03 | 7.7 | 0.04 ± 0.01 | 0.18 ± 0.08 | 350.0 | | 0.18 |
| Nuts and Seeds | | 0.21 ± 0.03 | | 0.19 ± 0.04 | | -9.5 | 0.12 ± 0.01 | 0.26 ± 0.06^**^ | 116.7 | 0.15 ± 0.02 | 0.18 ± 0.04 | 20.0 | | 0.32 |

^1^ a ratio of family income to poverty threshold ^2^ Linearized standard error

^3^ Percent change from 1999-2000 to 2009-2010;

Asterisks indicate a statistical significance in trends in types of protein within a subgroup (^*^ *P* <0.05,^**^ *P*<0.01, ^***^ *P*<0.001)
