# Supplementary material for: Trends in types of protein in US adolescents and children: Results from the National Health and Nutrition Examination Survey 1999-2010
Source: PLoS One. 2020 Mar 26;15(3):e0230686. doi: 10.1371/journal.pone.0230686 (PMC7098572; doi:10.1371/journal.pone.0230686)
Supplement: S6 Table — (DOCX) [file pone.0230686.s006.docx]

S6 Table. Mean intake of different types of protein in US children and adolescents (2-19 years), stratified by number of people in a household, National Health and Nutrition Examination Survey 1999-2010

|  | Fewer than three | | | Three or more | | |  |
| --- | --- | --- | --- | --- | --- | --- | --- |
| Intake in grams of protein foods (g) per kg of body weight ± SE^2^ | | | | | | | |
|  | 1999-2000 | 2009-2010 | Percent change^2^ | 1999-2000 | 2009-2010 | Percent change^2^ |  |
|  | (n=65) | (n=67) |  | (n=1,636) | (n=1,953) |  |  |
|  | Children (2-<12 years of age) | | | | | | *P*-interaction |
| Beef | 1.40 ± 0.34 | 1.21 ± 0.27 | -13.6 | 1.38 ± 0.12 | 1.13 ± 0.07 | -53.6 | 0.31 |
| Pork | 0.65 ± 0.33 | 0.51 ± 0.08 | -21.5 | 0.76 ± 0.08 | 0.57 ± 0.04 | -25.0 | 0.24 |
| Lamb or goat | 0.01 ± 0.01 | 0.01 ± 0.01 | 0 | 0.01 ± 0.01 | 0.01 ± 0.01 | 0 | 0.94 |
| Chicken | 1.60 ± 0.52 | 0.84 ± 0.17^***^ | -47.5 | 0.95 ± 0.07 | 1.31 ± 0.07 | -21.1 | 0.07 |
| Turkey | 0.17 ± 0.08 | 0.14 ± 0.06 | -17.6 | 0.20 ± 0.03 | 0.26 ± 0.03 | 30.0 | 0.05 |
| All Poultry | 1.77 ± 0.57 | 0.98 ± 0.18^***^ | -44.6 | 1.16 ± 0.09 | 1.56 ± 0.08 | -24.1 | 0.005 |
| Fish and shellfish | 0.18 ± 0.11 | 0.40 ± 0.12 | 122.2 | 0.18 ± 0.03 | 0.21 ± 0.05 | 16.7 | 0.29 |
| Milk and Milk products | 16.25 ± 3.41 | 18.43 ± 2.66 | 13.4 | 17.28 ± 0.87 | 18.69 ± 0.5 | 8.2 | 0.40 |
| Eggs | 0.28 ± 0.05 | 0.87 ± 0.27^*^ | 210.7 | 0.64 ± 0.04 | 0.68 ± 0.04^**^ | 6.3 | 0.33 |
| Legumes | 0.20 ± 0.06 | 0.63 ± 0.25 | 215.0 | 0.35 ± 0.04 | 0.54 ± 0.07 | 54.3 | 0.11 |
| Nuts and Seeds | 0.49 ± 0.30 | 0.56 ± 0.24 | 14.3 | 0.45 ± 0.05 | 0.40 ± 0.03 | -11.1 | 0.63 |
| Adolescents (12-19 years of age) | | | | | | | |
|  | 1999-2000 | 2009-2010 | Percent change^2^ | 1999-2000 | 2009-2010 | Percent change^2^ |  |
|  | (n=166) | (n=95) |  | (n=2,053) | (n=1,170) |  |  |
| Beef | 0.96 ± 0.18 | 1.07 ± 0.17 | 11.5 | 0.91 ± 0.09 | 0.64 ± 0.06^**^ | -29.7 | 0.11 |
| Pork | 0.37 ± 0.08 | 0.25 ± 0.04 | -32.4 | 0.34 ± 0.03 | 0.42 ± 0.06 | 23.5 | 0.40 |
| Lamb or goat | 0.01 ± 0.01 | 0.01 ± 0.01 | 0 | 0.01 ± 0.01 | 0.02 ± 0.01 | 100.0 | 0.74 |
| Chicken | 0.54 ± 0.07 | 0.57 ± 0.13 | 5.6 | 0.60 ± 0.05 | 0.75 ± 0.06^**^ | 25.0 | 0.32 |
| Turkey | 0.10 ± 0.03 | 0.15 ± 0.06 | 50.0 | 0.13 ± 0.02 | 0.12 ± 0.01 | -7.7 | 0.43 |
| All Poultry | 0.65 ± 0.09 | 0.72 ± 0.16 | 10.8 | 0.73 ± 0.06 | 0.88 ± 0.06^**^ | 20.5 | 0.49 |
| Fish and shellfish | 0.09 ± 0.03 | 0.26 ± 0.10 | 188.9 | 0.12 ± 0.02 | 0.11 ± 0.02 | -8.3 | 0.36 |
| Milk and Milk products | 3.78 ± 1.07 | 3.67 ± 0.85 | -2.9 | 5.85 ± 0.31 | 5.45 ± 0.39 | -6.8 | 0.35 |
| Eggs | 0.23 ± 0.03 | 0.20 ± 0.05 | -13.0 | 0.29 ± 0.02 | 0.33 ± 0.04 | 13.8 | 0.90 |
| Legumes | 0.14 ± 0.01 | 0.10 ± 0.06 | -28.6 | 0.13 ± 0.03 | 0.16 ± 0.04 | 23.1 | 0.21 |
| Nuts and Seeds | 0.20 ± 0.07 | 0.26 ± 0.09 | 30.0 | 0.16 ± 0.01 | 0.21 ± 0.03 | 31.3 | 0.77 |

^1^ Linearized standard error

^2^ Percent change from 1999-2000 to 2009-2010

Asterisks indicate a statistical significance in trends in types of protein within a subgroup (^*^ *P* <0.05,^**^ *P*<0.01, ^***^ *P*<0.001)
