# Supplementary material for: Trends in types of protein in US adolescents and children: Results from the National Health and Nutrition Examination Survey 1999-2010
Source: PLoS One. 2020 Mar 26;15(3):e0230686. doi: 10.1371/journal.pone.0230686 (PMC7098572; doi:10.1371/journal.pone.0230686)
Supplement: S7 Table — (DOCX) [file pone.0230686.s007.docx]

S7 Table. Mean intake of different types of protein in US children and adolescents (2-19 years), stratified by SNAP participation, National Health and Nutrition Examination Survey 1999-2010

|  | SNAP participants | | | Income-eligible non-participants | | |  |
| --- | --- | --- | --- | --- | --- | --- | --- |
| Intake in grams of protein foods (g) per kg of body weight ± SE^1^ | | | | | | | |
|  | 1999-2000 | 2009-2010 | Percent change^2^ | 1999-2000 | 2009-2010 | Percent change^2^ |  |
|  | (n=633) | (n=895) |  | (n=995) | (n=537) |  |  |
| Children and Adolescents (2-19 years of age) | | | | | | | *P*-interaction |
| Beef | 1.28 ± 0.15 | 1.13 ± 0.07 | -11.7 | 1.16 ± 0.2 | 1.00 ± 0.17 | -13.8 | 0.66 |
| Pork | 0.73 ± 0.08 | 0.63 ± 0.06 | -13.7 | 0.61 ± 0.08 | 0.53 ± 0.12 | -13.1 | 0.72 |
| Lamb or goat | 0.04 ± 0.01 | 0.01 ± 0.01 | -75.0 | 0.01 ± 0.001 | 0.002 ± 0.001 | -80.0 | 0.15 |
| Chicken | 0.92 ± 0.17 | 1.33 ± 0.10^*^ | 44.6 | 0.78 ± 0.1 | 1.09 ± 0.17^*^ | 39.7 | 0.28 |
| Turkey | 0.28 ± 0.06 | 0.24 ± 0.04 | -14.3 | 0.13 ± 0.02 | 0.24 ± 0.05^**^ | 84.6 | 0.01 |
| All Poultry | 1.20 ± 0.21 | 1.58 ± 0.11 | 31.7 | 0.91 ± 0.1 | 1.33 ± 0.18^**^ | 46.2 | 0.39 |
| Fish and shellfish | 0.16 ± 0.05 | 0.12 ± 0.02 | -25.0 | 0.25 ± 0.08 | 0.19 ± 0.05 | -24.0 | 0.55 |
| Milk and Milk products | 12.37 ± 0.78 | 14.22 ± 0.86 | 15.0 | 11.15 ± 0.94 | 11.4 ± 0.62 | 2.2 | 0.64 |
| Eggs | 0.63 ± 0.09 | 0.65 ± 0.09 | 3.2 | 0.53 ± 0.07 | 0.49 ± 0.05 | -7.5 | 0.62 |
| Legumes | 0.33 ± 0.03 | 0.38 ± 0.05 | 15.2 | 0.26 ± 0.06 | 0.27 ± 0.06 | 3.8 | 0.75 |
| Nuts and Seeds | 0.21 ± 0.04 | 0.32 ± 0.03 | 52.4 | 0.35 ± 0.07 | 0.29 ± 0.07 | -17.1 | 0.49 |

^1^ Linearized standard error

^2^ Percent change from 1999-2000 to 2009-2010

SNAP, Supplemental Nutrition Assistance Program.

Asterisks indicate a statistical significance in trends in types of protein within a subgroup (^*^ *P* <0.05,^**^ *P*<0.01, ^***^ *P*<0.001)
