# Supplementary material for: Trends in types of protein in US adolescents and children: Results from the National Health and Nutrition Examination Survey 1999-2010
Source: PLoS One. 2020 Mar 26;15(3):e0230686. doi: 10.1371/journal.pone.0230686 (PMC7098572; doi:10.1371/journal.pone.0230686)
Supplement: S8 Table — (DOCX) [file pone.0230686.s008.docx]

S8 Table. Mean intake of different types of protein in US children and adolescents (5-<19 years), stratified by NSLP and SBP participation, National Health and Nutrition Examination Survey 1999-2010

|  | NSLP and SBP participants | | | Income-eligible non-participants | | |  |
| --- | --- | --- | --- | --- | --- | --- | --- |
|  | Intake in grams of protein foods (g) per kg of body weight ± SE^1^ | | | | | | |
|  | 1999-2000 | 2009-2010 | Percent change^2^ | 1999-2000 | 2009-2010 | Percent change^2^ |  |
|  | (n=1,127) | (n=1,039) |  | (n=511) | (n=292) |  |  |
|  | Children and Adolescents (5-<19 years of age) | | | | | | *P*-interaction |
| Beef | 1.03 ± 0.18 | 0.89 ± 0.06 | -13.6 | 0.82 ± 0.09 | 0.86 ± 0.16 | 4.9 | 0.81 |
| Pork | 0.67 ± 0.07 | 0.61 ± 0.08 | -9.0 | 0.51 ± 0.09 | 0.35 ± 0.06 | -31.4 | 0.52 |
| Lamb or goat | 0.01 ± 0.01 | 0.01 ± 0.004 | 0 | 0.002 ± 0.002 | 0.06 ± 0.04 | 2900.0 | 0.32 |
| Chicken | 0.76 ± 0.08 | 1.10 ± 0.12^**^ | 44.7 | 0.55 ± 0.06 | 0.62 ± 0.09^**^ | 12.7 | 0.04 |
| Turkey | 0.20 ± 0.05 | 0.16 ± 0.03 | -20.0 | 0.13 ± 0.03 | 0.25 ± 0.04 | 92.3 | 0.05 |
| All Poultry | 0.96 ± 0.12 | 1.26 ± 0.12^**^ | 31.3 | 0.68 ± 0.07 | 0.87 ± 0.10^**^ | 27.9 | 0.18 |
| Fish and shellfish | 0.15 ± 0.05 | 0.14 ± 0.03 | -6.7 | 0.20 ± 0.05 | 0.10 ± 0.03 | -50.0 | 0.06 |
| Milk and Milk products | 9.94 ± 0.48 | 9.63 ± 0.54 | -3.1 | 8.75 ± 1.08 | 8.89 ± 0.70 | 1.6 | 0.71 |
| Eggs | 0.46 ± 0.07 | 0.51 ± 0.06 | 10.9 | 0.4 ± 0.08 | 0.36 ± 0.06 | -10.0 | 0.32 |
| Legumes | 0.19 ± 0.04 | 0.29 ± 0.05 | 52.6 | 0.14 ± 0.05 | 0.21 ± 0.06 | 50.0 | 0.02 |
| Nuts and Seeds | 0.19 ± 0.03 | 0.27 ± 0.02 | 42.1 | 0.36 ± 0.13 | 0.18 ± 0.03 | -50.0 | 0.20 |

^1^ Linearized standard error

^2^ Percent change from 1999-2000 to 2009-2010

NSLP, National School Lunch Program; SBP, School Breakfast Program.

Asterisks indicate a statistical significance in trends in types of protein within a subgroup (^*^ *P* <0.05,^**^ *P*<0.01, ^***^ *P*<0.001)
