# Supplementary material for: Trends in types of protein in US adolescents and children: Results from the National Health and Nutrition Examination Survey 1999-2010
Source: PLoS One. 2020 Mar 26;15(3):e0230686. doi: 10.1371/journal.pone.0230686 (PMC7098572; doi:10.1371/journal.pone.0230686)
Supplement: S9 Table — (DOCX) [file pone.0230686.s009.docx]

S9 Table. Mean intake of different types of protein in US among children and adolescents, National Health and Nutrition Examination Survey 1999-2010, adjusting for total energy intake instead of body weight

|  | 1999-2000 | 2009-2010 | Percent change^2^ |  |
| --- | --- | --- | --- | --- |
|  | (n=3,920) | (n=3,285) |  |  |
| Intake in grams of protein foods (g) ± SE^1^ | | | | *P-*trend |
|  | Children (2-<12 years of age) | |  |  |
| Beef | 33.0 ± 2.8 | 28.5 ± 2.1 | -13.6 | 0.53 |
| Pork | 18.9 ± 1.6 | 14.4 ± 0.8 | -23.8 | 0.23 |
| Lamb or goat | 0.3 ± 0.1 | 0.5 ± 0.3 | 66.7 | 0.54 |
| Chicken | 23.8 ± 1.5 | 31.2 ± 1.7 | 31.1 | <0.001 |
| Turkey | 4.8 ± 0.7 | 6.04 ± 0.6 | 25.8 | 0.13 |
| All poultry | 28.7 ± 2.0 | 37.3 ± 2.0 | 30.0 | <0.001 |
| Fish and shellfish | 4.5 ± 0.8 | 5.1 ± 0.8 | 13.3 | 0.88 |
| Milk and Milk products | 389.7 ± 17.7 | 412.8 ± 10.0 | 5.9 | 0.80 |
| Eggs | 14.6 ± 0.9 | 16.3 ± 0.9 | 11.6 | 0.01 |
| Legumes | 7.2 ± 0.8 | 11.9 ± 1.4 | 65.3 | 0.004 |
| Nuts and Seeds | 10.4 ± 1.3 | 9.6 ± 0.74 | -7.7 | 0.63 |
|  | Adolescents (12-19 years of age) | |  |  |
| Beef | 56.9 ± 5.3 | 43.5 ± 2.7 | -23.6 | 0.08 |
| Pork | 21.4 ± 1.6 | 25.2 ± 2.9 | 17.8 | 0.39 |
| Lamb or goat | 0.8 ± 0.4 | 1.1 ± 0.7 | 37.5 | 0.79 |
| Chicken | 35.9 ± 3.3 | 47.3 ± 3.3 | 31.8 | 0.001 |
| Turkey | 7.6 ± 0.9 | 7.6 ± 0.7 | 0 | 0.35 |
| All poultry | 43.4 ± 3.4 | 55.0 ± 3.5 | 26.7 | 0.001 |
| Fish and shellfish | 6.7 ± 1.2 | 7.6 ± 1.3 | 13.4 | 0.41 |
| Milk and Milk products | 335.6 ± 22.3 | 323.9 ± 20.4 | -3.5 | 0.59 |
| Eggs | 17.2 ± 0.9 | 20.6 ± 2.1 | 19.8 | 0.31 |
| Legumes | 7.6 ± 1.7 | 9.8 ± 1.9 | 28.9 | 0.31 |
| Nuts and Seeds | 10.3 ± 1.1 | 13.4 ± 1.9 | 30.1 | 0.06 |

^1^ Linearized standard error

^2^ Percent change from 1999-2000 to 2009-2010
