# Supplementary material for: Trends in types of protein in US adolescents and children: Results from the National Health and Nutrition Examination Survey 1999-2010
Source: PLoS One. 2020 Mar 26;15(3):e0230686. doi: 10.1371/journal.pone.0230686 (PMC7098572; doi:10.1371/journal.pone.0230686)
Supplement: S10 Table — (DOCX) [file pone.0230686.s010.docx]

S10 Table. Mean intake of types of protein in US adolescents and children for individuals with two days of dietary recalls

|  | 2003-2004 | 2005-2006 | 2007-2008 | 2009-2010 | Percent change^2^ |  |
| --- | --- | --- | --- | --- | --- | --- |
|  | Intake in grams per kg of body weight (g/kg) ± SE for the overall study population^1^ | | | |  | *P* trend |
| 2-<12 years of age | | | | | | |
| Beef | 1.30±0.06 | 1.18±0.06 | 1.32±0.12 | 1.16±0.08 | -10.8 | 0.37 |
| Pork | 0.61±0.03 | 0.68±0.06 | 0.63±0.03 | 0.60±0.04 | -1.6 | 0.60 |
| Lamb or goat | 0.02±0.01 | 0.01±0.01 | 0.01±0.01 | 0.005±0.001 | -100.0 | 0.13 |
| Chicken | 1.25±0.06 | 1.13±0.05 | 1.26±0.06 | 1.35±0.08 | 8.0 | 0.18 |
| Turkey | 0.25±0.02 | 0.19±0.01 | 0.26±0.02 | 0.26±0.02 | 4.0 | 0.19 |
| All poultry | 1.50±0.05 | 1.32±0.06 | 1.52±0.07 | 1.62±0.09 | 8.0 | 0.10 |
| Fish and shellfish | 0.19±0.03 | 0.28±0.07 | 0.17±0.02 | 0.24±0.07 | 26.3 | 0.52 |
| Milk and Milk products | 19.20±0.76 | 18.42±0.51 | 17.24±0.49 | 18.97±0.46 | -1.2 | 0.85 |
| Eggs | 0.79±0.06 | 0.75±0.05 | 0.68±0.04 | 0.73±0.03 | -7.6 | 0.26 |
| Legumes | 0.51±0.09 | 0.67±0.11 | 0.65±0.10 | 0.54±0.06 | 5.9 | 0.88 |
| Nuts and Seeds | 0.41±0.04 | 0.39±0.03 | 0.37±0.03 | 0.40±0.03 | -2.4 | 0.78 |
| 12-19 years of age | | | | | | |
| Beef | 0.86±0.04 | 0.83±0.04 | 0.73±0.07 | 0.77±0.07 | -10.5 | 0.17 |
| Pork | 0.38±0.04 | 0.35±0.02 | 0.33±0.03 | 0.39±0.04 | 2.6 | 0.98 |
| Lamb or goat | 0.01±0.003 | 0.004±0.001 | 0.01±0.002 | 0.01±0.01 | 0.0 | 0.74 |
| Chicken | 0.62±0.03 | 0.68±0.04 | 0.73±0.04 | 0.74±0.05 | 19.4 | 0.03 |
| Turkey | 0.14±0.01 | 0.13±0.01 | 0.15±0.03 | 0.14±0.02 | 0.0 | 0.89 |
| All poultry | 0.77±0.03 | 0.81±0.03 | 0.88±0.05 | 0.88±0.05 | 14.3 | 0.03 |
| Fish and shellfish | 0.13±0.02 | 0.14±0.02 | 0.14±0.02 | 0.11±0.01 | -15.4 | 0.34 |
| Milk and Milk products | 5.94±0.40 | 5.53±0.25 | 5.23±0.27 | 5.37±0.36 | -9.6 | 0.23 |
| Eggs | 0.29±0.02 | 0.27±0.01 | 0.31±0.03 | 0.31±0.02 | 6.9 | 0.29 |
| Legumes | 0.16±0.02 | 0.15±0.02 | 0.15±0.02 | 0.17±0.03 | 6.3 | 0.72 |
| Nuts and Seeds | 0.19±0.02 | 0.19±0.02 | 0.21 ± 0.03 | 0.19±0.02 | 0.0 | 0.83 |

^1^ g/kg indicates grams of protein food intake per kilogram of body weight, and SE indicates standard errors.

^2^ Percent change from 1999-2000 to 2009-2010
